# A methodological framework for evaluating transitions in acute care services in the Netherlands to achieve Triple Aim

**DOI:** 10.1186/s13104-022-06187-w

**Published:** 2022-09-09

**Authors:** Rosa Naomi Minderhout, Mattijs E. Numans, Hedwig M. M. Vos, Marc A. Bruijnzeels

**Affiliations:** Department of Public Health and Primary Care/HealthCampus The Hague, Leiden University Medical Centre, Turfmarkt 99, 5thflourflour, 2511 DP The Hague, the Netherlands

**Keywords:** Triple aim, Population health management, Acute care services, Integrated care, Evaluate, Crowding

## Abstract

**Objective:**

The accessibility of acute care services is currently under pressure, and one way to improve services is better integration. Adequate methodology will be required to provide for a clear and accessible evaluation of the various intervention initiatives. The aim of this paper is to develop and propose a Population Health Management(PHM) methodology framework for evaluation of transitions in acute care services.

**Results:**

Our methodological framework is developed from several concepts found in literature, including Triple Aim, integrated care and PHM, and includes continuous monitoring of results at both project and population levels. It is based on a broad view of health rather than focusing on a specific illness and facilitates the evaluation of various intervention initiatives in acute care services in the Netherlands and distinctly explains every step of the evaluation process and can be applied to a heterogeneous group of patients.

**Supplementary Information:**

The online version contains supplementary material available at 10.1186/s13104-022-06187-w.

## Introduction

Acute care services are currently overstretched [1–3]. In the Dutch setting, acute care services involves many different organisations, including Emergency Departments (EDs), General Practice Cooperatives (GPCs), ambulance services, acute mental health services, and home care and nursing home organisations^4^]. Overcrowding is caused by a combination of factors such as the growing influx of patients due to the ageing population combined with a shortage of healthcare personnel [1–3], the suboptimal use of acute care services as a high proportion of acute care service use is by patients with low-urgency problems [5, 6], and the fragmentation of acute care services with an absence of system-wide coordination and planning [7]. Acute care services need to integrate in order to improve care [8]. Unfortunately, a general framework to assess the impact of interventions is lacking. In this paper we develop a methodological framework to assess the impact of solutions to increase the efficacy of acute care services.

There are several frameworks that focus on the integration of care services, but these do not exclusively emphasise acute care services. The more comprehensive international population health value perspective has been summarised in the Triple Aim. Triple Aim defines improvement of a healthcare system as the simultaneous pursuit of three linked aims: improving the individual experience of care, improving the health of populations, and reducing healthcare cost growth [9, 10]. The experience of care professionals also plays an important role, and adds a fourth aspect, which increasingly often leads towards the ‘Quadruple Aim’ to be reached in healthcare reform initiatives [11].

Based on the Triple Aim approach, we present a new methodological framework specifically designed to evaluate acute care initiatives. Diverse initiatives can be evaluated within this framework in a consistent manner, which should lead to improvements in our understanding of current problems faced during the provision of acute care services. The researchers have already used this framework for the evaluation about the value of merging medical data from ambulance services and GPCs [12].

## Main text

### Methods: several concepts

#### Triple Aim and learning system

**Fig. 1:**
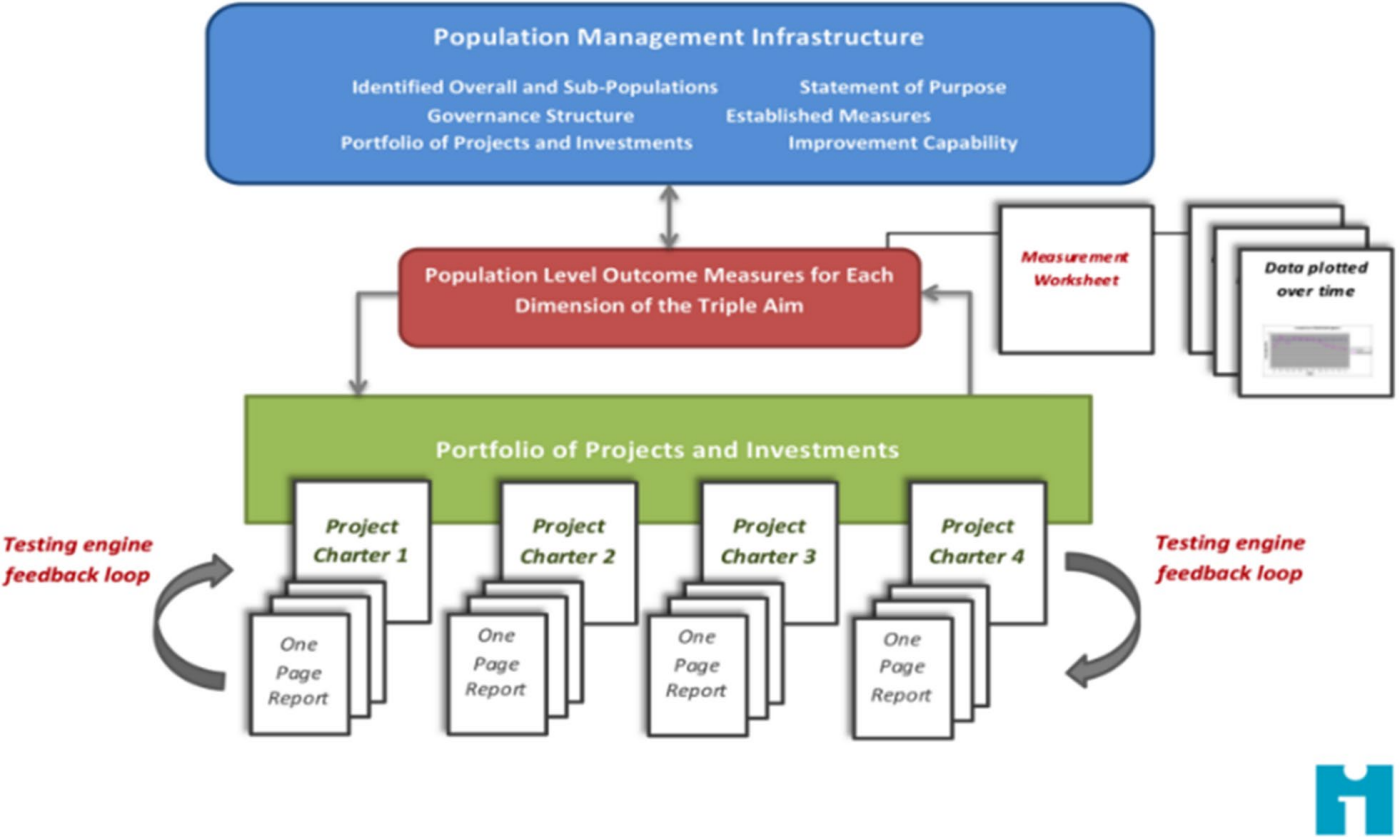
Triple Aim infrastructure by the Institute for Healthcare Improvement

The Triple Aim infrastructure (Fig. 1) [10] focuses on aspects on two levels: the population level and the project level. At the population level, the infrastructure focuses on population aims such as quality of care, population health and per capita cost. A common purpose regarding a specific population that encourages the collaboration amongst all stakeholders is very important [9]. At the project level, organisations need to collaborate with a focus on the project goals through a portfolio of projects and investments, each project and investment contributes partly to the population goals. Different types of integrations can be implemented within the same population. The framework encourages improvement through a ‘learning system’ in which newly acquired knowledge is used to develop and adopt changes that improve performance [9]. To safeguard a learning system, projects include structured evaluation moments to monitor the progress of improvement.

#### Integrated care

**Fig. 2:**
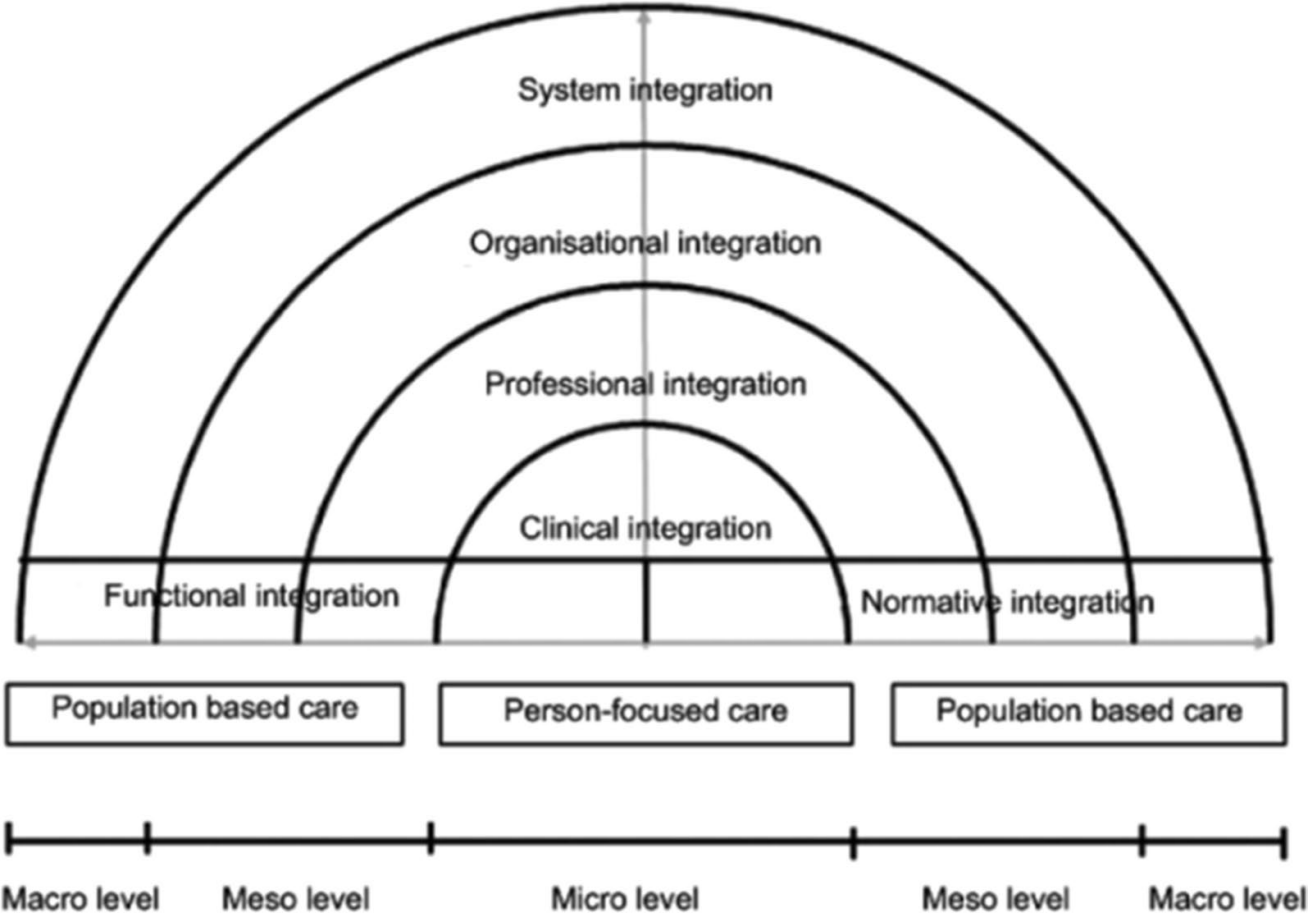
Rainbow model for integrated care (RMIC)

Since integration of services is one of the key elements in the acute care initiatives the second concept concerns integrated care. To achieve a better understanding of integrated care initiatives, the researchers adopted the Rainbow model for integrated care (RMIC) [13]. The RMIC is a conceptual framework to improve insight into the six interrelated dimensions of integrated care: clinical, professional, organizational, systems, functional, and normative integration (Fig. 2) [14, 15]. These dimensions play complementary roles on the micro-, meso- and macrolevels. At the micro level, where the patient meets the professional, all professionals cooperate to provide the best care for the individual. At the meso level, professionals and organisations need to align processes, services and interests to facilitate the micro level. Finally, at the macro level the government and funders should stimulate system integration. Functional and normative integration should ensure the linking of the micro, meso and macro levels with the system. Functional integration includes planning, human resource-, information- and financial-management. Normative integration includes a shared mission, vision and culture [14]. In the Triple Aim infrastructure the project level should be linked with the population level through normative and functional integration [13].

#### Population health management

Population Health Management (PHM) refers to the large-scale transformation required for the reorganisation and integration of different services at all levels of integration. These efforts cover public health, healthcare, social care and wider public services with the aim of improving outcomes, and are summarized in the Triple Aim [16]. The concept of PHM is still developing but some care principles are: [17]

- Assessment of an adverse event
- Define a specific population with a high risk of adverse outcomes in healthcare (a burning platform)
- Stratify the population according to the risk of the adverse event
- Apply proactive interventions tailored to the needs of the specific risk adjusted subgroups

## Results: methodological tools

Five steps for the evaluation of acute care services came out of merging the principles and key elements of the three concepts and synthesising the commonalities.

### Step 1: Identification of the specific population

What is the burning platform within that population that needs action? According to literature, an example of a specific population that will potentially derive maximum benefit from improvement in acute services can be older patients with comorbidities. Older adults often make increased use of acute care and some studies have reported an approximately fivefold higher rate of emergency admissions among patients aged 70 years or above compared to patients aged 30 years or less [18, 19]. This group also experiences higher rates of adverse outcomes such as return to the ED, hospitalisation or death [20]. Due to comorbidities, the elderly often receive help from multiple healthcare, social care and home care providers and are therefore subject to care fragmentation as a result of poor coordination of services and a lack of communication between care providers [21].

### Step 2: Triple Aim outcomes

#### 2a: Population health outcomes

To evaluate the health outcomes of a population, it is important to establish clear indicators. Indicators consist of all possible ‘care pathways’ that a patient with an acute care request may use. The amount of care use gives an impression of health status. Therefore, indicators for population health outcomes in a framework specific for acute care services include ED visits, hospitalisation, use of the ambulance service, use of the GPC, contact with the GP, psychiatric care, home care or transfer to a nursing home, and death after an acute care request during the follow-up period.

### Step 2b: Per capita cost

Within these indicators for population health outcomes, per capita costs of healthcare should be estimated and taken into account, see Table 1.

**Table 1:** Costs made in acute care services

Indicators	
Emergency care visits	Cost incurred during an emergency visit during a follow-up period
Hospitalisation	Costs incurred during hospital stay during follow-up period
GP use	Costs of the number of times that a patient reports with an acute request for help from the GP during a follow-up period
Psychiatric care	Cost of the number of times that a patient reports to the crisis service with an acute request and any hospitalization costs during a follow-up period
Nursing home	Cost of the number of days a patient is admitted to the nursing home after acute care request during a follow-up period
Ambulance service	Cost of the number of times that a patient has been transported with the ambulance after an acute treatment request during a follow-up period
Home care	Cost of the number of hours a patient needed a district nurse after acute treatment request during a follow-up period
Short stay nursing homes	Cost of the number of days a patient is admitted to a first-line stay after acute care request during a follow-up period

### Step 2c: Experience of care

#### Patient experiences

To assess experiences with acute care services during a project, questionnaires can be completed by the patients receiving care. Examples include Consumer Assessment of Healthcare Providers and Systems (CAHPS) and How’s Your Health [9]. The Consumer Quality Index (CQI) questionnaires (CQI Emergency department, CQI ambulance care and CQI GPC) [22, 23], which is based on the CAHPS, is often used in the Netherlands.

#### Experiences of healthcare professionals

Job satisfaction and wellbeing are of considerable importance as they both help protect healthcare professionals against somatic complaints, psychological distress and burnout [24]. This is especially important in acute services since healthcare workers in this area are more prone to burn-out due to their frontline roles with patients, the large turnover rate of patients, and the lack of a buffer [25]. The experiences of professionals can be evaluated with questionnaires, an example is the validated Leiden Quality of Work Life Questionnaire [26].

### Step 3: Follow-up period

The continuous monitoring of results at the population level plotted over time provides an impression of longitudinal outcomes. To determine the results of a service we have to first choose a follow-up period. In literature, the length of the follow-up period per case in a specific population varies from 14 days to 12 months, with outcomes at 30 and 90 days being the most commonly reported follow-up periods [27, 28].

### Step 4: a comparable group

In order to demonstrate a difference between regular care and care during pilot projects in acute services, data on a comparable group from the specific population is also needed. When a large project is undertaken there is often no comparison group available. In this case patient data before commencement of the project (baseline measurements) can be used. This allows researchers to examine some outcome of interest prior to an intervention, but it does not eliminate the possibility that results within the intervention group might have occurred regardless of the intervention [29].

### Step 5: a learning system

To ensure a learning system, all projects include some structured evaluation moments that allow monitoring of the progress of improvements. The researcher may release some interim project results during structured evaluations but this should not interfere with possible improvements. The ‘Integration monitor Care coordination’ (see Additional file 1: appendix A), based on the validated RMIC Measurement Tool [30], was developed by the researchers to measure care coordination and was applied to acute care projects in the Netherlands but has not been validated yet.

## Discussion

In current literature, more and more studies arise that describe the implementation of transformation initiatives, [16, 31–33] but providers generally struggled due to a lack of guidance and an absence of composite sets of measurements that allow performance assessment [34]. As available data generally lacks clarity regarding the selection and implementation of purposeful measures, the researchers hope that this new framework will fill this gap for acute care services. This framework shows the necessity of a mixed-methods approach in which we combine the epidemiologic rigor of a pragmatic cohort study with specific outcomes, follow up period and control situations and a more action research oriented approach of a learning system to assess the improvement of integration of services as a determinant of the Triple Aim outcomes. The three goals must be achieved simultaneously. Organisations have a collective responsibility to ensure the health and well-being of a population by taking shared responsibility for the integration of services. A collective, shared perspective on the evaluation and interpretation of the results makes negotiations easier and allows goals to be pursued in this multi-stakeholder network [10].

Evaluating similar acute care initiatives in an unambiguous manner in order to compare results and make adjustments where necessary is of considerable added value. Small-scale successes must be promoted so that a common language can be established and successful initiatives can be implemented on a larger scale. To ensure a learning system, projects must include structured evaluation moments that allow monitoring of the progress of improvements, preferably based on routinely registered data. This requires statistics at the regional level that are discussed within the network and are used to encourage adjustments. This method of evaluation differs from a more managerial blueprint implementation in which everything is recorded in advance. This methodological framework permits the evaluation of various acute care initiatives in practice and ensures that initiatives can be evaluated consistently so that the general body of knowledge will improve. The Integration monitor Care coordination is a good example of practical application. Instruments used in evaluation must provide insight into practical issues, validity is certainly important but adjustments are sometimes necessary to make it work in practice. The outcome is a more or less circular process that facilitates the continuous improvement [15]. This new framework helps to share experiences and learnings between projects while still ongoing, thus promoting a learning system. This addresses one of the key challenges today, when having a major reform going on with multiple projects, that they all face similar challenges but need to come up with solutions separately because there is no common framework—and even if there is, like the Innovation fund in Germany [35], project progress is assessed individually and not compared.

## Conclusions

The new framework was developed based on literature and was designed to help in the assessment of project outcomes in acute services carried out by different healthcare organisations. We recommend that those involved in setting up a project in an acute care organisation consider applying this framework, since it enhances the comparability of mechanisms and outcomes.

## Limitations

- The validity has yet to be determined in practice
- During projects in the Netherlands it emerged that data collection in acute care services was very demanding in view of the multiple organisations that need to cooperate within the multi-stakeholder network [12].
- As there is a diversity in the acute care settings in the industrialized world, any framework should be adapted to country and region-specific factors.

## Supplementary Information

**Additional file 1:** **Appendix A**. Example of an integration meter questionnaire. This ‘Integration monitor care coordination’ was used in the Netherlands.

## Data Availability

Not applicable.

## Abbreviations

CAHPS: Consumer Assessment of Healthcare Providers and Systems
CQI: Consumer Quality Index
ED: Emergency department
GPC: General practice cooperative
GP: General practitioner
IHI: Institute for Healthcare Improvement
LUMC: Leiden University Medical Centre
PHM: Population Health Management
RMIC: Rainbow model for integrated care

## References

[1] Moskop JC, Sklar DP, Geiderman JM, Schears RM, Bookman KJ (2009). Emergency department crowding, part 1–concept, causes, and moral consequences. Ann Emerg Med.

[2] Pines JM, Hilton JA, Weber EJ, Alkemade AJ, Al Shabanah H, Anderson PD (2011). International perspectives on emergency department crowding. Acad Emerg Med.

[3] Bittencourt RJ, Stevanato AM, Bragança C, Gottems LBD, O'Dwyer G (2020). Interventions in overcrowding of emergency departments: an overview of systematic reviews. Rev Saude Publica.

[4] Kroneman M, Boerma W, van den Berg M, Groenewegen P, de Jong J, van Ginneken E (2016). Netherlands: health system review. Health Syst Transit.

[5] Giesen P, Franssen E, Mokkink H, van den Bosch W, van Vugt A, Grol R (2006). Patients either contacting a general practice cooperative or accident and emergency department out of hours: a comparison. Emerg Med J: EMJ.

[6] Minderhout RNN, Venema P, Vos HMM, Kant J, Bruijnzeels MA, Numans ME (2019). Understanding people who self-referred in an emergency department with primary care problems during office hours: a qualitative interview study at a daytime general practice cooperative in two hospitals in The Hague, The Netherlands. BMJ Open.

[7] Taymour RK, Abir M, Chamberlin M, Dunne RB, Lowell M, Wahl K (2018). Policy, practice, and research agenda for emergency medical services oversight: a systematic review and environmental scan. Prehosp Disaster Med.

[8] McDonald KM, Sundaram V, Bravata DM, Lewis R, Lin N, Kraft SA, et al. Closing the Quality Gap: A Critical Analysis of Quality Improvement Strategies (Vol. 7: Care Coordination). Rockville (MD): Agency for Healthcare Research and Quality (US); 2007. https://www.ncbi.nlm.nih.gov/books/NBK44015/20734531

[9] Stiefel M, Nolan K. A guide to measuring the triple aim: population health, experience of care, and per capita cost. IHI Innovation Series white paper Cambridge, Massachusetts: Institute for Healthcare Improvement. 2012. https://www.ihi.org/

[10] Berwick DM, Nolan TW, Whittington J (2008). The triple aim: care, health, and cost. Health Aff.

[11] Bachynsky N (2020). Implications for policy: the triple aim, quadruple aim, and interprofessional collaboration. Nurs Forum.

[12] Minderhout RN, Vos HMM, van Grunsven PM, de la Torre YRI, Alkir-Yurt S, Numans ME (2021). The value of merging medical data from ambulance services and general practice cooperatives using triple aim outcomes. Int J Integr Care.

[13] Valentijn PP, Schepman SM, Opheij W, Bruijnzeels MA (2013). Understanding integrated care: a comprehensive conceptual framework based on the integrative functions of primary care. Int J Integr Care.

[14] Valentijn PP, Boesveld IC, van der Klauw DM, Ruwaard D, Struijs JN, Molema JJ (2015). Towards a taxonomy for integrated care: a mixed-methods study. Int J Integr Care.

[15] Boesveld IC, Bruijnzeels MA, Hitzert M, Hermus MA, van der Pal-de KM, van Den Akker-van MM (2017). Typology of birth centres in the Netherlands using the rainbow model of integrated care: results of the dutch birth centre study. BMC Health Serv Res.

[16] Steenkamer B, Drewes H, Putters K, van Oers H, Baan C (2020). Reorganizing and integrating public health, health care, social care and wider public services: a theory-based framework for collaborative adaptive health networks to achieve the triple aim. J Health Serv Res Policy.

[17] Steenkamer BM, Drewes HW, Heijink R, Baan CA, Struijs JN (2017). Defining population health management: a scoping review of the literature. Popul Health Manag.

[18] George G, Jell C, Todd B (2006). Effect of population ageing on emergency department speed and efficiency: a historical perspective from a district general hospital in the UK. Emerg Med J.

[19] Wass A, Zoltie N (1996). Changing patterns in accident and emergency attenders. Emerg Med J.

[20] Aminzadeh F, Dalziel WB (2002). Older adults in the emergency department: a systematic review of patterns of use, adverse outcomes, and effectiveness of interventions. Ann Emerg Med.

[21] Deschodt M, Laurent G, Cornelissen L, Yip O, Zuniga F, Denhaerynck K (2020). Core components and impact of nurse-led integrated care models for home-dwelling older people: a systematic review and meta-analysis. Int J Nurs Stud.

[22] Bos N, Sturms LM, Stellato RK, Schrijvers AJ, van Stel HF (2015). The consumer quality Index in an accident and emergency department: internal consistency, validity and discriminative capacity. Health Expect.

[23] Smirnova A, Lombarts K, Arah OA, van der Vleuten CPM (2017). Closing the patient experience chasm: a two-level validation of the consumer quality index inpatient hospital care. Health Expect.

[24] De Jonge J, Schaufeli WB (1998). Job characteristics and employee well-being: A test of Warr's Vitamin Model in health care workers using structural equation modelling. J Organiz Behav.

[25] Ifediora CO (2016). Burnout among after-hours home visit doctors in Australia. BMC Fam Pract.

[26] van der Doef M, Maes S (1999). The leiden quality of work questionnaire: its construction, factor structure, and psychometric qualities. Psychol Rep.

[27] Cousins G, Bennett Z, Dillon G, Smith SM, Galvin R (2013). Adverse outcomes in older adults attending emergency department: systematic review and meta-analysis of the triage risk stratification tool. Eur J Emerg Med.

[28] Galvin R, Gilleit Y, Wallace E, Cousins G, Bolmer M, Rainer T (2017). Adverse outcomes in older adults attending emergency departments: a systematic review and meta-analysis of the Identification of Seniors At Risk (ISAR) screening tool. Age Ageing.

[29] Salkind NJ. Encyclopedia of research design: SAGE Publications, Inc.; 2010. 10.4135/9781412961288

[30] Valentijn P, Angus L, Boesveld I, Nurjono M, Ruwaard D, Vrijhoef H (2017). Validating the rainbow model of integrated care measurement tool: results from three pilot studies in the Netherlands, Singapore and Australia. Int J Integr Care.

[31] Struijs JN, Drewes HW, Stein KV (2015). Beyond integrated care: challenges on the way towards population health management. Int J Integr Care.

[32] Suter E, Oelke ND, Adair CE, Armitage GD (2009). Ten key principles for successful health systems integration. Healthc Q.

[33] World Health Assembly. Framework on integrated, people-centred health services: report by the Secretariat. World Health Organization. 2016; 69. https://apps.who.int/iris/handle/10665/252698

[34] Obucina M, Harris N, Ja F, Chai A, Radford K, Ross A, Carr L, Vecchio N (2018). The application of triple aim framework in the context of primary healthcare: A systematic literature review. Health Policy..

[35] Berghöfer A, Göckler DG, Sydow J, Auschra C, Wessel L, Gersch M (2020). The German health care innovation fund–an incentive for innovations to promote the integration of health care. J Health Organiz Manage.

