# Supplementary material for: A methodological framework for evaluating transitions in acute care services in the Netherlands to achieve Triple Aim
Source: BMC Res Notes. 2022 Sep 9;15:296. doi: 10.1186/s13104-022-06187-w (PMC9463780; doi:10.1186/s13104-022-06187-w)
Supplement: Supplementary file 1 — Additional file 1: Appendix A. Example of an integration meter questionnaire. This ‘Integration monitor care coordination’ was used in the Netherlands. [file 13104_2022_6187_MOESM1_ESM.docx]

**Appendix A: Example of an integration meter questionnaire
This ‘Integration monitor Care coordination’ was used in the Netherlands**

| **Domain** | **Determinant** | **Niv** | **Description** |
| --- | --- | --- | --- |
| **Clinical integration** | Triage | 1 | Every organization does its own triage with a separate triage system |
|  |  | 2 | Every organization does its own triage but with an unambiguous triage system |
|  |  | 3 | There is a telephone consultation with high-risk patients between different organizations about the triage |
|  |  | 4 | One general triagist for different organizations together |
|  | Deployment of the right healthcare prof. | 1 | Different organizations are not considered together which healthcare professional can best be used |
|  |  | 2 | If a high-risk patient is involved, it is occasionally examined which  prof. can best be deployed. Consultation takes place by telephone |
|  |  | 3 | There is always a telephone consultation between the various profs if another prof. can be used more effectively |
|  |  | 4 | The various profess are present in one room and decide jointly which prof. can best be deployed |
|  | Transfer patient data | 1 | Profs never receive telephone or written patient data from the other organization |
|  |  | 2 | Profs receive patient data only by telephone transfer in case of a referral from another organization |
|  |  | 3 | Profs receive patient data both digitally and by telephone transfer in case of a referral from another organization |
|  |  | 4 | One ICT system with an electronic file is used for the entire care process |
|  | Case management | 1 | No coordinated policy, each prof. does this separately |
|  |  | 2 | Incidental policy is coordinated by several profs in high-risk patients |
|  |  | 3 | High-risk patients are regularly discussed in multidisciplinary consultation where policy is coordinated |
|  |  | 4 | All high-risk patients are discussed in multidisciplinary consultation where policy is coordinated |
|  | Outflow coordination | 1 | No cooperation with the organizations that facilitate outflow |
|  |  | 2 | Incidentally collaboration with organizations that facilitates outflow |
|  |  | 3 | Organizations are regularly involved in facilitating outflow |
|  |  | 4 | Fixed agreements exist with the organizations involved in the outflow of patients. A joint responsibility is felt. |
| **Professional integration** | Vision of healthcare profs | 1 | No common vision |
|  |  | 2 | A common vision, but it is not shared by everyone |
|  |  | 3 | A common vision that is propagated by all organizations |
|  |  | 4 | A common vision, also for the long term, where all organizations are held responsible for realizing the vision |
|  | Protocols | 1 | Every prof uses exclusively his own monodisciplinary protocols |
|  |  | 2 | Profs are aware of protocols from other disciplines, but do not use them themselves |
|  |  | 3 | Profs use multidisciplinary protocols. When a deviation of it is made, they do not discuss the reasons for this deviation |
|  |  | 4 | Profs use multidisciplinary protocols. When a deviation from the protocol is made, account is taken of the reasons for this deviation |
|  | Interprof. Education | 1 | Every organization arranges its own in-service training |
|  |  | 2 | Multidisciplinary training is offered to all organizations, but is not required |
|  |  | 3 | Multidisciplinary training is offered to all organizations. Some of them are mandatory (such as dealing with acute situations) |
|  |  | 4 | Multidisciplinary training with regard to urgent actions is required to be regularly followed with all organizations, as a fixed component of a common policy |
|  | Interprof. Governance | 1 | Results of the quality of care provided by profs are never discussed with the other organization |
|  |  | 2 | Results of the quality of care provided by profs are discussed occasionally |
|  |  | 3 | Results of the quality of care provided by profs are discussed structurally with each other, no further action is taken |
|  |  | 4 | Results of the quality of care provided are structurally discussed with each other and evaluated. They are jointly responsible for the entire care process. |
| **Organizational integration** | Learning organization | 1 | No evaluation or joint meetings with representatives of the different organizations |
|  |  | 2 | Occasional evaluation during meetings with representatives |
|  |  | 3 | structural evaluation during meetings with representatives and if necessary an improvement plan is drawn up |
|  |  | 4 | Structural evaluation takes place during meetings with representatives and an improvement plan is drawn up if necessary based on the results of evaluations + regularly monitored |
|  | Evaluate the acute care chain | 1 | Functioning of the acute care chain is never evaluated within the region |
|  |  | 2 | Functioning of the acute care chain is occasionally evaluated within the region; no fixed indicators have been drawn up for this |
|  |  | 3 | Functioning of the acute care chain is occasionally evaluated within the region on the basis of process and outcome indicators |
|  |  | 4 | Functioning of the acute care chain is structurally evaluated within the region on the basis of process and outcome indicators and directs the implementation of the acute care where necessary |
|  | Common objectives | 1 | No joint objectives have been set by the organizations |
|  |  | 2 | Joint objectives have been formulated, but these are not evaluated |
|  |  | 3 | Joint objectives have been formulated that are structurally measured and evaluated |
|  |  | 4 | A joint objective has been formulated that will be evaluated and monitored on a structural basis. Active action is taken to achieve the objectives |
|  | Complaints regulations | 1 | Every discipline has its own complaints regulations |
|  |  | 2 | In the case of a complaint, the total care process is being looked into |
|  |  | 3 | Complaints are picked up and processed jointly |
|  |  | 4 | One complaints regulations exists for the total care process |
|  | Service management | 1 | Professionals have no shared service management with each other |
|  |  | 2 | Profs share project facilities jointly (e.g. a joint information leaflet) |
|  |  | 3 | Prof. delen structureel gemeenschappelijke faciliteiten en iedere organisatie handhaaft zijn eigen faciliteiten |
|  |  | 4 | Profs have shared structural facilities and each organization maintains its own facilities (e.g. there is a joint website, but every discipline also has its own website) |
| **Functional integration** | Information management | 1 | information systems of the various profs are not integrated |
|  |  | 2 | Information systems of the various profs are partially integrated or accessible to different care providers |
|  |  | 3 | information systems of the various profs form one system |
|  |  | 4 | Information systems of the various profs form one system and are accessible to the patient |
|  | Feedback with quality indicators | 1 | Profs within the acute care chain never evaluate each other's performance with the help of quality indicators |
|  |  | 2 | Profs within the acute care chain evaluate each other's performance incidentally with the help of joint quality indicators |
|  |  | 3 | Profs within the acute care chain inform and discuss each other's performance structurally on the basis of national quality indicators |
|  |  | 4 | Profs share common and national quality indicators for the entire care process. These indicators are structurally evaluated and the implementation of the acute care is adjusted where necessary |
| **System integration** | Environment management | 1 | Many different organizations in the region, such as general practitioners, ambulance services and district nurse organizations, and this inhibits cooperation |
|  |  | 2 | Many different organizations in the region but this does not play a role in the cooperation |
|  |  | 3 | Many different organizations in the region and this facilitates good cooperation |
|  |  | 4 | The acute service act as one organization (with multiple disciplines) |
|  | Cooperation with health insurers | 1 | Insurers are not involved in the project |
|  |  | 2 | Insurance companies are occasionally involved |
|  |  | 3 | Insurers are structurally involved by the organizations and joint interests are discussed |
|  |  | 4 | Insurers are part of the various stakeholders involved. There are structural meetings to discuss each other's interests and objectives. The objectives are regularly evaluated and monitored |
|  | Cooperation with the Ministry of Health inspection | 1 | Inspection is not involved |
|  |  | 2 | Inspection is occasionally involved |
|  |  | 3 | Inspection is structurally involved by the organizations and joint interests are discussed |
|  |  | 4 | Inspection is part of the various stakeholders involved. There are structural meetings to discuss each other's interests and objectives. The objectives are regularly evaluated and monitored |
|  | Cooperation with acute care in the region | 1 | Acute care network is not involved |
|  |  | 2 | Acute care network is occasionally involved |
|  |  | 3 | Acute care network is structurally involved by the organizations and joint interests are discussed |
|  |  | 4 | Acute care network is part of the various stakeholders involved. There are structural meetings to discuss each other's interests and objectives. The objectives are regularly evaluated and monitored |
|  | Cooperation with patient associations and client council | 1 | Patient associations or client councils are not involved |
|  |  | 2 | Patient associations or client councils are occasionally involved |
|  |  | 3 | Patient associations or client councils are structurally involved by the organizations and joint interests are discussed |
|  |  | 4 | Patient associations or client councils are part of the various stakeholders involved. There are structural meetings to discuss each other's interests and objectives. The objectives are regularly evaluated and monitored |
| **Normative integration** | Trust | 1 | No trust between the various chain partners |
|  |  | 2 | Little confidence in chain partners |
|  |  | 3 | Trust between the chain partners; they know each other well and can trust everyone |
|  |  | 4 | Trust between the chain partners; attention is paid to maintaining trust by making it regularly negotiable |
|  | Visionary leadership | 1 | No profs within the various organizations with a vision about cooperation |
|  |  | 2 | A few profs within the various organizations with a vision about cooperation |
|  |  | 3 | Profs within the various organizations with a vision about   cooperation that inspires and enthuses cooperation |
|  |  | 4 | Profs with a vision about cooperation that inspires and thus motivates and encourages collaboration and action. As a result, the different organizations become more and more a whole |
|  | Informal cooperation | 1 | Every organization has its own culture and the informal contacts remain within their own organization |
|  |  | 2 | Every organization knows its own culture and conscious activities are regularly developed to get to know each other |
|  |  | 3 | There are good mutual informal relationships, but cultural differences are still noticeable |
|  |  | 4 | There is a culture which the mutual informal relationships between all organizations are excellent |

**References**
The following references have been used for the composition of the Integrationmonitor Care coordination:

- Valentijn PP, Schepman SM, Opheij W, Bruijnzeels MA. Understanding integrated care: a comprehensive conceptual framework based on the integrative functions of primary care. International Journal of Integrated Care 2013;13.
- Dimensies van geïntegreerde eerstelijnszorg. www.jvei.nl/wp-content/uploads/Dimensies-Van-Ge%C3%AFntegreerde-Eerstelijnszorg-II.pdf. Verschenen in De Eerstelijns, maart 2012. (verkregen 05-08-2016).
- Berwick DM, Nolan TW, Whittington J. The triple aim: care,health, and cost. Health Aff (Millwood) 2008 May-Jun;27(3):759-769.
- Boesveld, I. C., Annegarn, A. M. A., IJsseldijk, J. M., Veldhuyzen, D. C., Winkel, L., Annot, F., & Wiegers, T. A. (2016). Geïntegreerde geboortezorg in VSV’s: resultaten van de VSV-Integratiemeter.
- Kwaliteitskader Spoedzorgketen. https://www.zorginzicht.nl/bibliotheek/kwaliteitskader-spoedzorgketen/KwaliteitsstandaardenDocumenten/Kwaliteitskader%20Spoedzorgketen.pdf. Zorginstituut Nederland, januari 2018
- AZN (2017) Ambulancezorg in 2025: Zorgcoördinatie en mobiele zorg. Visiedocument Ambulancezorg Nederland. Juli 2017.
- Broek F. van den, Grunsven P. van, Giesen P., Smits M. (2014) Afstemming huisarts en ambulance dringend nodig – Minder vervoer en meer behandeling ter plekke zijn mogelijk. Medisch Contact. December 2014.
